# Unraveling the Evolutionary History of Snow Sheep (*Ovis nivicola*): Genome‐Wide Single Nucleotide Polymorphism Analysis Reveals Genetic Diversity and Clarifies Taxonomy

**DOI:** 10.1002/ece3.74182

**Published:** 2026-08-14

**Authors:** Arsen V. Dotsev, Innokentiy M. Okhlopkov, Mikhail G. Bondar, Dennis I. Litovka, Tatiana E. Deniskova, Veronika R. Kharzinova, Michael N. Romanov, Taras P. Sipko, Nikolai V. Mamaev, Maria N. Semerikova, Olga A. Koshkina, Neckruz F. Bakoev, Vugar A. Bagirov, Andrey A. Sitsko, Dmitry G. Medvedev, Alexander N. Kuksin, Mohammad Hossein Moradi, Ali Esmailizadeh, Darren K. Griffin, Henry Reyer, Klaus Wimmers, Gábor Mészáros, Johann Sölkner, Ivica Medugorac, Gottfried Brem, Natalia A. Zinovieva

**Affiliations:** ^1^ L. K. Ernst Federal Research Center for Animal Husbandry Podolsk Russia; ^2^ Institute of Biological Problems of the Cryolithozone Siberian Branch of the Russian Academy of Sciences Yakutsk Russia; ^3^ Joint Directorate of Taimyr Reserves Norilsk Russia; ^4^ Chukotka Arctic Research Center Anadyr Russia; ^5^ School of Natural Sciences University of Kent Canterbury UK; ^6^ Animal Genomics and Bioresource Research Unit (AGB Research Unit), Faculty of Science Kasetsart University Bangkok Thailand; ^7^ Severtsov Institute of Ecology and Evolution Russian Academy of Sciences Moscow Russia; ^8^ Rosokhotrybolovsoyuz Association Moscow Russia; ^9^ Department of Game Management and Bioecology Irkutsk State University of Agriculture Molodezhny Russia; ^10^ Tuva Institute for the Integrated Development of Natural Resources of the Siberian Branch of the Russian Academy of Sciences Kyzyl Russia; ^11^ Department of Animal Science University of Tehran Karaj Iran; ^12^ Department of Animal Science Shahid Bahonar University of Kerman Kerman Iran; ^13^ EGA Institute for Women's Health, Preimplantation Genetics Group University College London London UK; ^14^ Research Institute for Farm Animal Biology (FBN) Dummerstorf Germany; ^15^ Division of Livestock Sciences University of Natural Resources and Life Sciences Vienna Austria; ^16^ Population Genomics Group, Department of Veterinary Sciences LMU Munich Munich Germany; ^17^ Institute of Animal Breeding and Genetics University of Veterinary Medicine (VMU) Vienna Austria

**Keywords:** artiodactyl genomics, conservation genetics, genetic differentiation, population structure, Russian Far East, subspecies validation

## Abstract

The snow sheep ( *Ovis nivicola* ) is a wild mountain artiodactyl inhabiting the highlands of northeastern Siberia and the Russian Far East. Despite its ecological and conservation importance, the intraspecific taxonomy of this species has long remained controversial due to its broad distribution, morphological variability and limited available material. In this study, we performed a comprehensive genome‐wide single nucleotide polymorphism (SNP) analysis, encompassing all major described populations to clarify the evolutionary relationships and taxonomic structure of snow sheep. A total of 337 individuals from 15 populations were genotyped using Illumina OvineSNP50 and OvineHD 600 K BeadChips, with 1032 autosomal SNPs retained after quality filtering. Population structure analyses revealed eight major genetic clusters largely corresponding to geographic regions: Kamchatkan, Koryak, Okhotsk/Chukotka, Yakutian (Moma Range), Yakutian (Verkhoyansk Range), Yablonov, Kharaulakh and Putorana. Pairwise *F*_ST_ values confirmed substantial differentiation among most populations. Using f_3_‐statistics, two significant admixture events were identified, one involving the Orulgan and another the northern Kamchatka populations. Based on the integrated genomic and historical evidence, our results support the taxonomic recognition of the Kamchatkan (*O. n. nivicola*), Putorana (*O. n. borealis*), Okhotsk (*O. n. alleni*), Koryak (*O. n. koriakorum*), Yakutian (*O. n. lydekkeri*), and Stanovoy/Yablonov (*O. n. potanini*) subspecies, and propose recognition of the Kharaulakh snow sheep as a new subspecies, *O. n. ernsti*. The Kodar population, characterized by extremely low heterozygosity, requires further sampling to resolve its taxonomic position. These findings provide the first genome‐wide framework for understanding the evolutionary history of snow sheep and establish a genetic basis for future conservation management.

## Graphical abstract

This study chronicles all described subspecies of snow sheep ( *Ovis nivicola* ) since 1829, reviewing taxonomic assessments by key systematists. Using genome‐wide SNP genotyping on 337 samples from 15 populations, we validate and refine subspecies classifications, revealing eight genetic clusters and proposing a new subspecies (*O. n. ernsti*). Our molecular approach integrates historical and genomic evidence for conservation insights.

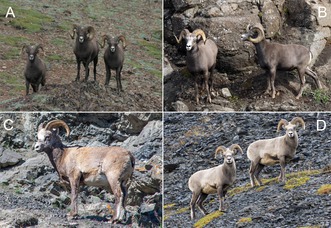

## 1 Introduction

**FIGURE 1:**
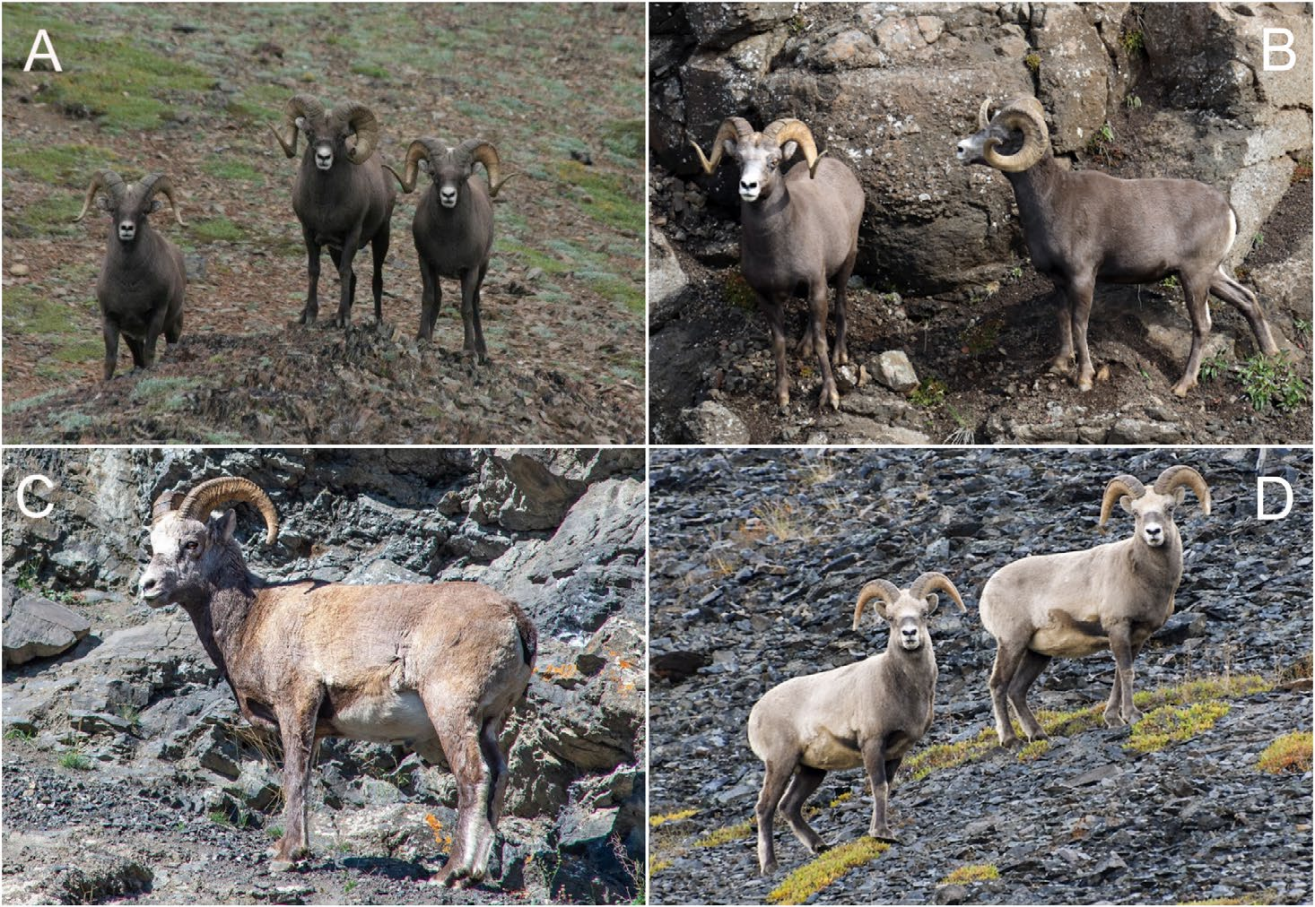
Representatives of the snow sheep ( *Ovis nivicola* ) from different regions: (A) Kamchatka Krai, Kronotsky Nature Reserve (photo by Yu.A. Yarovenko; CC‐BY‐4.0 open license, https://rusmam.ru/data/view?id=52361); (B) Putorana Plateau (photo by M.G. Bondar); (C) Yakutia, Suntar‐Khayata Range (photo by A. Krivoshapov; CC‐BY‐4.0 open license, https://rusmam.ru/data/view?id=31031); (D) Yakutia, Kharaulakh Range (photo by N. Protodiakonov & S. Ponomarenko).

The snow sheep ( *Ovis nivicola* Eschscholtz 1829) (Figure 1) is a wild artiodactyl species distributed across the mountain systems of northeastern Siberia and the Russian Far East. As a large‐bodied herbivore, it plays an important ecological role in high‐altitude ecosystems and holds considerable conservation significance, while also representing a valuable game species. Phylogenetically, it is closely related to the North American wild species, that is, bighorn sheep ( *O. canadensis* Shaw 1804) and thinhorn sheep ( *O. dalli* Nelson 1884), all classified within the subgenus *Pachyceros* (Geist 1971; Valdez 1982). Although interspecific relationships within the genus *Ovis* are relatively well resolved, the intraspecific taxonomy of snow sheep remains controversial and ambiguous. That is, there is ongoing debate regarding the number and validity of described subspecies. The latter, for example, concerns the putative nature of subspecies *O. n*. *alleni*, *O. n*. *kodarensis* and *O. n*. *potanini* (Damm and Franco 2014; Bürglin 2025). Valdez (1982) acknowledged the validity of only *O. n*. *nivicola* and *O. n*. *borealis*. The Integrated Taxonomic Information System (ITIS 2025) recognizes four subspecies: *O. n*. *borealis*, *O. n. kodarensis*, *O. n*. *koriakorum*, and *O. n. nivicola*. Carruthers (1949) listed five subspecies: *O. n*. *nivicola* (= *storcki*), *O. n*. *borealis*, *O. n*. *alleni*, *O. n*. *lydekkeri*, and *O. n*. *potanini*. Other classifications take into consideration up to six subspecies (as reviewed by Bunch et al. 2006): *O. n*. *koriakorum*, *O. n*. *nivicola*, *O. n*. *alleni*, *O. n*. *potanini*, *O. n*. *borealis*, and *O. n*. *lydekkeri*.

Historically, morphological traits and habitat (population range) were primary classification criteria and the basis for proposing approximately 10 snow sheep species/subspecies throughout the 19th and 20th centuries (Nasonov 1923; Tsalkin 1951; Sokolov 1959; Heptner et al. 1961; Revin et al. 1988; Zheleznov‐Chukotsky 1994; Medvedev 1994; Chernyavsky 2004). Concurrently, an alternative viewpoint suggested that subspecies should not be distinguished at all because of their high degree of morphological similarity; instead the reclassification of the snow sheep as a subspecies of *O. canadensis* was proposed (Danilkin 2005). Due to the remote distribution of snow sheep and the scarcity of available material, taxonomic descriptions were often based on a single specimen. For example, in 1904 Joel Asaph Allen described a new species, *O. storcki*, from a skull found near Fort Tigil on Kamchatka, noting that its horns more closely resembled those of the argali ( *O. ammon* ) rather than *O. nivicola* . It was later demonstrated, however, that this represented only an age‐related variation within snow sheep (Nasonov 1923). A further limitation of morphology‐based classification is its sensitivity to environmental factors, including climatic conditions, disease, and nutrition. As a result, genetically related individuals may exhibit markedly different phenotypes, complicating reliable taxonomic delineation.

Consequently, molecular approaches, including mitochondrial DNA analysis, microsatellites, and genome‐wide single nucleotide polymorphism (SNP) data, have provided new opportunities for a more objective and rigorous assessment of intraspecific structure and phylogenetic relationships in wild animal populations. These approaches have already led to significant taxonomic revisions in Caprinae and related groups. For example, Ropiquet and Hassanin (2005) demonstrated that the genus *Hemitragus* is polyphyletic, which resulted in the reassignment of the Himalayan, Nilgiri, and Arabian tahrs to three distinct genera. Similarly, Rezaei et al. (2010) used mitochondrial and nuclear DNA markers to refine relationships within the genus *Ovis*. In the case of snow sheep, a mitochondrial DNA–based comparative analysis suggested that this species diverged from other members of the genus *Ovis* approximately 2.3 million years ago (Bunch et al. 2006). It was also proposed that the population inhabiting the Kharaulakh Range should be recognized as a distinct subspecies (Dotsev et al. 2021).

Among modern molecular approaches, genome‐wide SNP genotyping based on arrays developed for related domestic species has proven particularly effective for studying the genetic diversity and taxonomic structure of wild artiodactyls. High‐density SNP arrays originally designed for domestic animals have been successfully applied to closely related wild species, making them a valuable tool for investigating population structure, genetic diversity and taxonomy. For example, the Illumina PorcineSNP60 BeadChip, designed for domestic pigs, was effectively used to study wild boar populations and their hybridization with their domestic counterparts (Yang et al. 2017). Moreover, the BovineSNP50 BeadChip has been employed in genetic studies of European bison ( *Bison bonasus* ) (Tokarska et al. 2009) and reindeer ( *Rangifer tarandus* ) (Kharzinova et al. 2018; Kharzinova et al. 2020). Similarly, the GoatSNP50 BeadChip has been applied to wild goat species ( *Capra ibex* , *C. aegagrus* , *C. falconeri*, *C. pyrenaica* and *C. caucasica* ) to investigate population structure and to detect introgression from domestic goats (Pogorevc et al. 2024). The OvineSNP50 BeadChip has been particularly widely used: it enabled population genomic studies of North American wild sheep ( *O. canadensis* and *O. dalli* ) (Miller et al. 2011; Sim et al. 2016; Miller et al. 2018; Sim and Coltman 2019), snow sheep ( *O. nivicola* ) (Dotsev et al. 2018; Dotsev et al. 2026), argali ( *O. ammon* ) (Dotsev et al. 2023), Asiatic mouflon and urial ( *O. orientalis* , *O. vignei* ) (Dotsev et al. 2025), as well as the European mouflon ( *O. aries musimon* ) (Ciani et al. 2015). These examples clearly demonstrate that cross‐species application of SNP arrays is a powerful approach for artiodactyl genomics and provides essential insights into their taxonomy and conservation.

Building on this framework, the present study aimed to resolve taxonomic uncertainties and assess genetic diversity across available snow sheep ( *O. nivicola* ) populations using a genome‐wide SNP analysis approach. To achieve this, we first review the historical research on the intraspecific structure of snow sheep. For each subspecies ever described (in chronological order), we outline its taxonomic history, the opinions of prominent systematists, and how our genomic results contribute clarity to resolving ongoing debates. Rather than simply characterizing the genetic profiles of selected groups—as is common in such studies—we systematically evaluate all historically proposed subspecies and provide our diagnostic conclusions based on the SNP data.

## 2 Materials and Methods

### 2.1 Sample Collection

**FIGURE 2:**
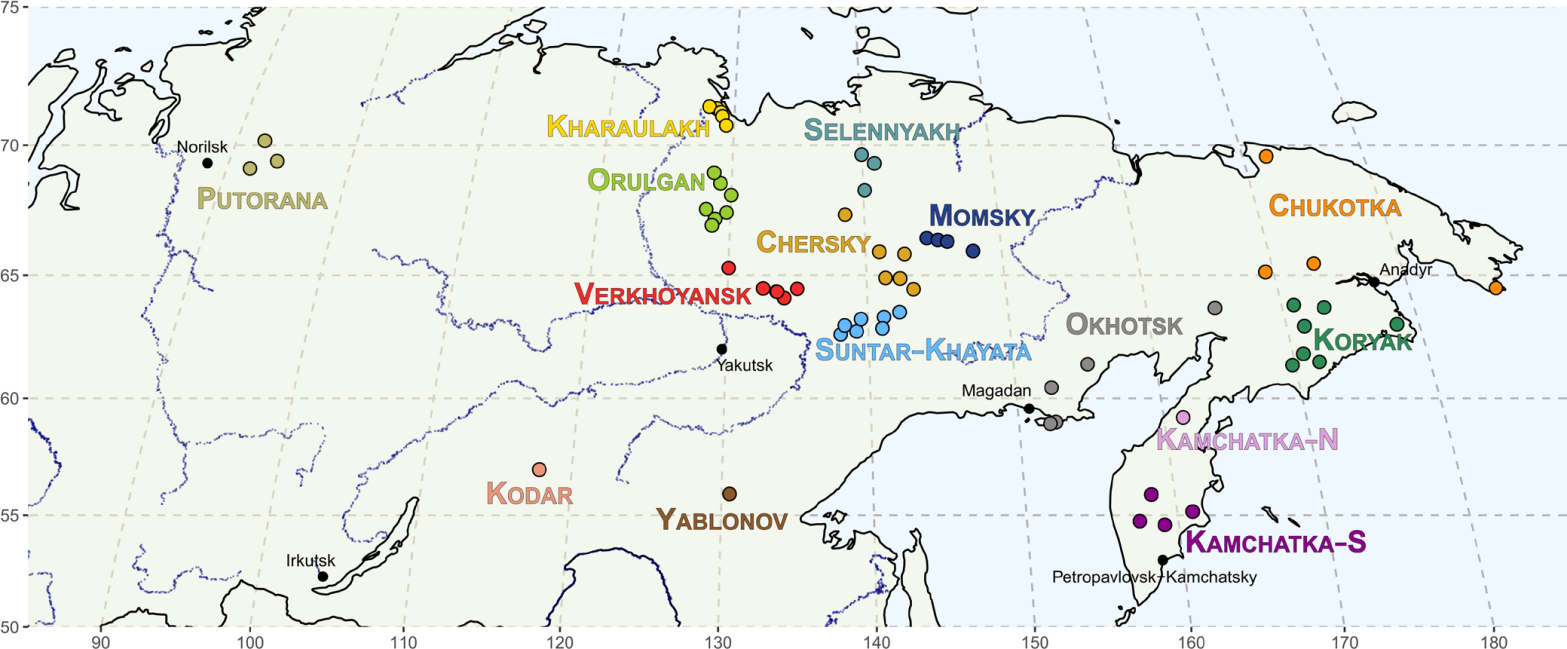
Sampling locations of the snow sheep ( *Ovis nivicola* ) populations studied.

A total of 337 snow sheep ( *O. nivicola* ) specimens, representing 15 populations, were examined in this study. Samples from the Kamchatka Peninsula were divided into two groups: Southern (Kamchatka‐S, *n* = 18) and Northern (Kamchatka‐N, *n* = 6). The Koryak subspecies (*O. n. koriakorum*) was represented by samples from the Koryak Mountains (Koryak, *n* = 37). Samples collected in the Chukotka Autonomous Okrug were classified as Chukotkan sheep (Chukotka, *n* = 7). The Okhotsk snow sheep (*O. n. alleni*) was represented by samples from the Magadan Oblast (Okhotsk, *n* = 22). Specimens collected in the Stanovoy Range were designated as Yablonov (Yablonov, *n* = 6). Specimens from Yakutia, traditionally classified as Yakutian snow sheep (*O. n. lydekkeri*), were divided into seven groups based on their mountain ranges: Verkhoyansk (*n* = 38), Momsky (*n* = 12), Kharaulakh (*n* = 41), Chersky (*n* = 19), Selennyakh (*n* = 7), Suntar‐Khayata (*n* = 45), and Orulgan (*n* = 72). Samples of the Putorana subspecies (*O. n. borealis*) were obtained from the Putorana Plateau (Putorana, *n* = 6). The Kodar snow sheep was represented by a single specimen (Kodar, *n* = 1). Details of sampling, including population designations, geographic locations, sample sizes and references, are summarized in Table 1, with corresponding sampling sites shown in Figure 2.

**TABLE 1:** Sampling information for the snow sheep ( *Ovis nivicola* ) and argali ( *O. ammon* ) populations studied.

Population	Region	*n*		References
		50 K + 600 K	600 K	
Kamchatka‐S	Eastern range (Kamchatka), Sredinny range (southern part)	18	8	Dotsev et al. (2026) and this study
Kamchatka‐N	Sredinny range (northern part)	6	4	This study
Koryak	Koryak mountains	37	10	Dotsev et al. (2026) and this study
Chukotka	Chukotka mountains, Anadyr highlands	7	5	Dotsev et al. (2026)
Okhotsk	Kolyma mountains	22	8	Dotsev et al. (2026) and this study
Momsky	Moma (Momsky) range	12	6	Dotsev et al. (2018) and this study
Selennyakh	Selennyakh range	7	7	This study
Chersky	Chersky range	19	9	This study
Suntar_Khayata	Suntar‐Khayata range	45	10	Dotsev et al. (2018) and this study
Yablonov	Stanovoy range	6	5	This study
Verkhoyansk	Central Verkhoyansk range	38	10	Dotsev et al. (2018) and this study
Orulgan	Orulgan range	72	10	Dotsev et al. (2018) and this study
Kharaulakh	Kharaulakh range	41	10	Dotsev et al. (2018) and this study
Putorana	Putorana plateau	6	3	This study
Kodar	Kodar mountains	1	—	This study
Altai argali (*O. a. ammon*)	Altai Mountains, Russia	—	6	Dotsev et al. (2023)
Pamir argali (*O. a. polii*)	Pamir Mountains, Tajikistan	—	8	Dotsev et al. (2023)

*Note: n*, number of samples; 50 K + 600 K, samples genotyped with both Illumina OvineSNP50 BeadChip and Illumina OvineHD BeadChip; 600 K, samples genotyped exclusively with Illumina OvineHD BeadChip.

The samples were collected during scientific expeditions and obtained from legally permitted trophy hunters. Specimens from protected (Red‐listed) populations were sourced exclusively from non‐harvested mortalities, including natural predator kills and accidental deaths. The study protocol (No. 2, 28 April 2022) was approved by the Ethics Commission for Animal Experiments of the L.K. Ernst Federal Research Center for Animal Husbandry.

### 2.2 DNA Extraction, Genotyping and Data Analysis

Muscle tissue samples were preserved in 96% ethanol and stored at −20°C until further processing. Genomic DNA was isolated using Nexttec columns (Nexttec Biotechnology GmbH, Leverkusen, Germany) according to the manufacturer's protocol. DNA concentration was measured with a Qubit 4.0 Fluorometer using the dsDNA HS Assay Kit (Thermo Fisher Scientific, Waltham, MA, USA), and DNA integrity was verified by electrophoresis on a 1% agarose gel.

Genotyping of the examined samples was performed using two Illumina (San Diego, CA, USA) Ovine BeadChips: the OvineSNP50 BeadChip and the Ovine Infinium HD SNP BeadChip 600 K, which contain approximately 50,000 and 600,000 SNPs, respectively. Only autosomal loci present in both arrays with genotype call (GC) and genotype train (GT) scores ≥ 0.5 were retained for subsequent analyses.

To filter out loci deviating from Hardy–Weinberg equilibrium (*p* < 0.001), we applied PLINK 1.9 (Purcell et al. 2007) (‐‐hwe 1E‐3) separately to each studied population. Following merging of all groups, we conducted additional quality control by excluding SNPs with genotyping rates < 90% (‐‐geno 0.1), those with minor allele frequency (MAF) < 1% (‐‐maf 0.01), and variants in linkage disequilibrium (LD; ‐‐indep‐pairwise 50 5 0.2). All samples included in the analyses had individual‐level genotyping call rates ≥ 90% (‐‐mind 0.1). Following these quality control steps, 1032 SNPs were retained for downstream analyses.

Principal component analysis (PCA) was performed using PLINK 1.9 and the results were visualized with ggplot2 R package (Wickham 2009). Genetic ancestry was estimated using ADMIXTURE v1.3 (Alexander et al. 2009) evaluating K‐values from 1 to 15. The optimal K‐value was determined by identifying the minimum cross‐validation error. The resulting ancestry proportions were plotted using pophelper package (Francis 2017) in R. Pairwise *F*_ST_ genetic distances were computed using the R package StAMPP (Pembleton et al. 2013). A phylogenetic network was then constructed from the *F*_ST_ distance matrix using the Neighbor‐Net algorithm implemented in SplitsTree6 (version 6.4.17) (Huson and Bryant 2024). Genetic diversity was assessed through metrics computed in diveRsity R package (Keenan et al. 2013): observed heterozygosity (*H*_O_) per locus, unbiased expected heterozygosity (_*U*_ *H*_*E*_), rarefied allelic richness (*A*_*R*_), and population‐specific inbreeding coefficients (*F*_IS_) with 95% confidence intervals. Rarefaction was applied to allelic richness to account for unequal sample sizes among populations, whereas unbiased expected heterozygosity was used to reduce bias associated with small sample sizes. Individual multilocus heterozygosity (MLH) was calculated as the proportion of heterozygous loci to total genotyped loci using the inbreedR package (Stoffel et al. 2016) in R. To assess potential population admixture, we computed Patterson's f_3_‐statistics (Patterson et al. 2012) using the admixr package (Petr et al. 2019) in R. To infer historical gene flow patterns, we reconstructed maximum‐likelihood population graphs using TreeMix v1.13 (Pickrell and Pritchard 2012). The optimal number of migration events was determined by evaluating the proportion of variance explained, assessed using the OptM R package (Fitak 2021) and residual plots for model fit diagnostics. To maximize genomic resolution, the f_3_ and TreeMix analyses were restricted to a dataset of 105 samples genotyped exclusively with the Illumina OvineHD BeadChip high‐density array: Kamchatka‐S (*n* = 8), Kamchatka‐N (*n* = 4), Koryak (*n* = 10), Chukotka (*n* = 5), Okhotsk (*n* = 8), Momsky (*n* = 6), Selennyakh (*n* = 7), Chersky (*n* = 9), Suntar‐Khayata (*n* = 10), Yablonov (*n* = 5), Verkhoyansk (*n* = 10), Orulgan (*n* = 10), Kharaulakh (*n* = 10), and Putorana (*n* = 3). An additional 14 samples of the argali representing two subspecies, Altai ( *O. ammon ammon* , *n* = 6) and Pamir ( *O. ammon polii* , *n* = 8), were included as an outgroup (Table 1). For the f3‐statistics and TreeMix analyses, a separate QC procedure was applied, resulting in a dataset of 9855 SNPs. The geographic distribution of sampling locations was visualized using R with the maps (Becker et al. 2018) and ggplot2 packages.

## 3 Results

### 3.1 Genetic Population Structure and Admixture

**FIGURE 3:**
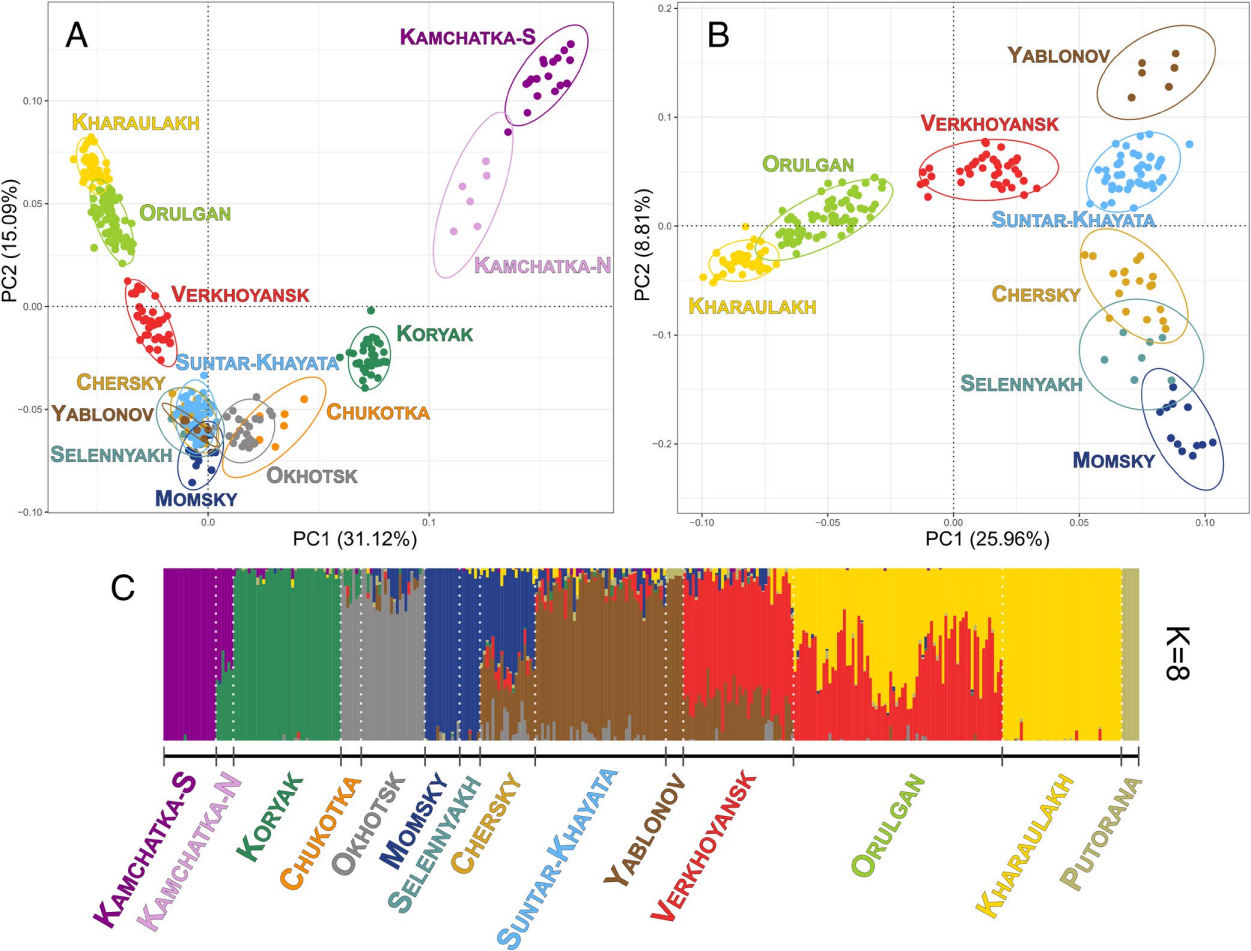
Genetic variation and ancestry in *Ovis nivicola* populations. (A) Principal component analysis (PCA) including all studied populations (PC1–PC2). (B) Principal component analysis (PCA) of populations from Yakutia and the Stanovoy Range (Yablonov) (PC1–PC2). (C) Individual ancestry proportions inferred with ADMIXTURE (*K* = 8). Circles represent 95% confidence ellipses for each population in the PCA.

To assess the genetic structure and variation among the major populations, we performed PCA, excluding the Putorana and Kodar groups, as their small sample sizes, geographic isolation and extremely low heterozygosity could distort clustering patterns and bias the interpretation of the results (Figure 3A and Figure S1A). The first three principal components (PC1, PC2 and PC3) accounted for 31.12%, 15.09% and 9.68% of the total variance, respectively. All individuals clustered according to their source populations, with no outliers detected. PCA results revealed clear genetic structuring, with Kamchatka‐S, Kamchatka‐N, Koryak, Verkhoyansk, Orulgan, and Kharaulakh each forming distinct clusters, indicating strong population differentiation. Chukotka and Okhotsk clustered together, while all remaining populations grouped into a single unified cluster.

Since some of these populations formed a tight cluster on the global PCA, a separate analysis was performed using a dataset restricted to samples from Yakutia and the Stanovoy Range (Yablonov) (Figure 3B and Figure S1B). The analysis reaffirmed the distinctness of the Kharaulakh population from other Yakutian snow sheep, as it was clearly separated along the PC1 axis in both the PC1–PC2 and PC1–PC3 projections. Similarly, the Central Verkhoyansk group formed a separate cluster that showed no overlap with any other populations. Furthermore, the Yablonov population was distinguished from *O. n. lydekkeri*, showing a clear separation from this subspecies specifically on the PC1–PC3 plot.

To examine population structure, estimate ancestry proportions and identify potential hybridization events among the studied groups, we conducted the ADMIXTURE analysis (Figure 3C). The optimal number of ancestral components, determined by cross‐validation (CV) error (Figure S2), was 8 (*K* = 8). At *K* = 8, it was found that their own ancestral components included the Kamchatka‐S, Koryak, Chukotka/Okhotsk, Momsky/Selennyakh, Yablonov/Suntar‐Khayata, Verkhoyansk, Kharaulakh, and Putorana populations. Kamchatka‐N had genetic components from Kamchatka‐S and Koryak, while Orulgan had contributions from the Verkhoyansk and Kharaulakh populations. At *K* = 2, the first two genetic components corresponded to the Kamchatka‐S and Kharaulakh ancestral types (Figure S3). The Momsky/Selennyakh component emerged at *K* = 3, followed by Putorana, Koryak, Verkhoyansk, Yablonov/Suntar‐Khayata, and Chukotka/Okhotsk at higher K‐values.

Furthermore, to investigate signals of admixture, we applied f_3_‐statistics. Two cases of significant admixture were identified (Table 2), involving the Orulgan and Kamchatka‐N populations. These findings are consistent with the ADMIXTURE results that also revealed mixed ancestry components in both groups. In both cases, the negative f_3_ values with high Z‐scores (−8.81 for Orulgan and −5.07 for Kamchatka‐N) provided strong evidence for admixture.

**TABLE 2:** Significant f_3_ statistics indicating admixture in *Ovis nivicola* populations.

A (Source)	B (Source)	C (Target)	f_3_	stderr	Z‐score	nsnps
Kharaulakh	Verkhoyansk	Orulgan	−0.010572	0.001200	−8.812	7487
Kamchatka_S	Koryak	Kamchatka_N	−0.018651	0.003678	−5.071	6768

*Note:* A (Source), 1st source population; B (Source), 2nd source population; C (Target), target population; f_3_, value for f_3_‐statistics; stderr, standard error; Z‐score, f_3_/stderr significant values (Z‐score < −3) indicating evidence of admixture in population C; nsnps, number of SNPs used for calculation.

### 3.2 Genetic Divergence and Evolutionary History

To measure genetic differentiation among the studied groups, pairwise *F*_ST_ genetic distances were estimated (Table S1). The resulting values ranged from 0.036 between the Chukotka and Okhotsk populations, indicating close genetic affinity, to 0.645 between the Putorana and Kamchatka‐S populations. Putorana showed the greatest similarity to Suntar‐Khayata (0.381) and Verkhoyansk (0.401). Kamchatka‐S exhibited the lowest differentiation from Koryak (0.248). Furthermore, Koryak was genetically close to its neighboring groups, with *F*_ST_ values of 0.113 to Okhotsk and 0.109 to the Chukotka population, respectively. We observed low levels of genetic differentiation between the Okhotsk and Yakutian snow sheep populations, with *F*_ST_ values ranging from 0.057 (Chersky) to 0.122 (Selennyakh). The genetic distance between Yablonov and Okhotsk, previously grouped with one another by many researchers (Tsalkin 1951; Heptner et al. 1961; Chernyavsky 1984), was relatively high (0.169), indicating that they should not be considered a single unit. The Kharaulakh sheep exhibited genetic distances (*F*_ST_) with other Yakutian populations ranging from 0.101 (Verkhoyansk) to 0.212 (Momsky), with a notably low value of 0.039 observed only for the admixed Orulgan population.

**FIGURE 4:**
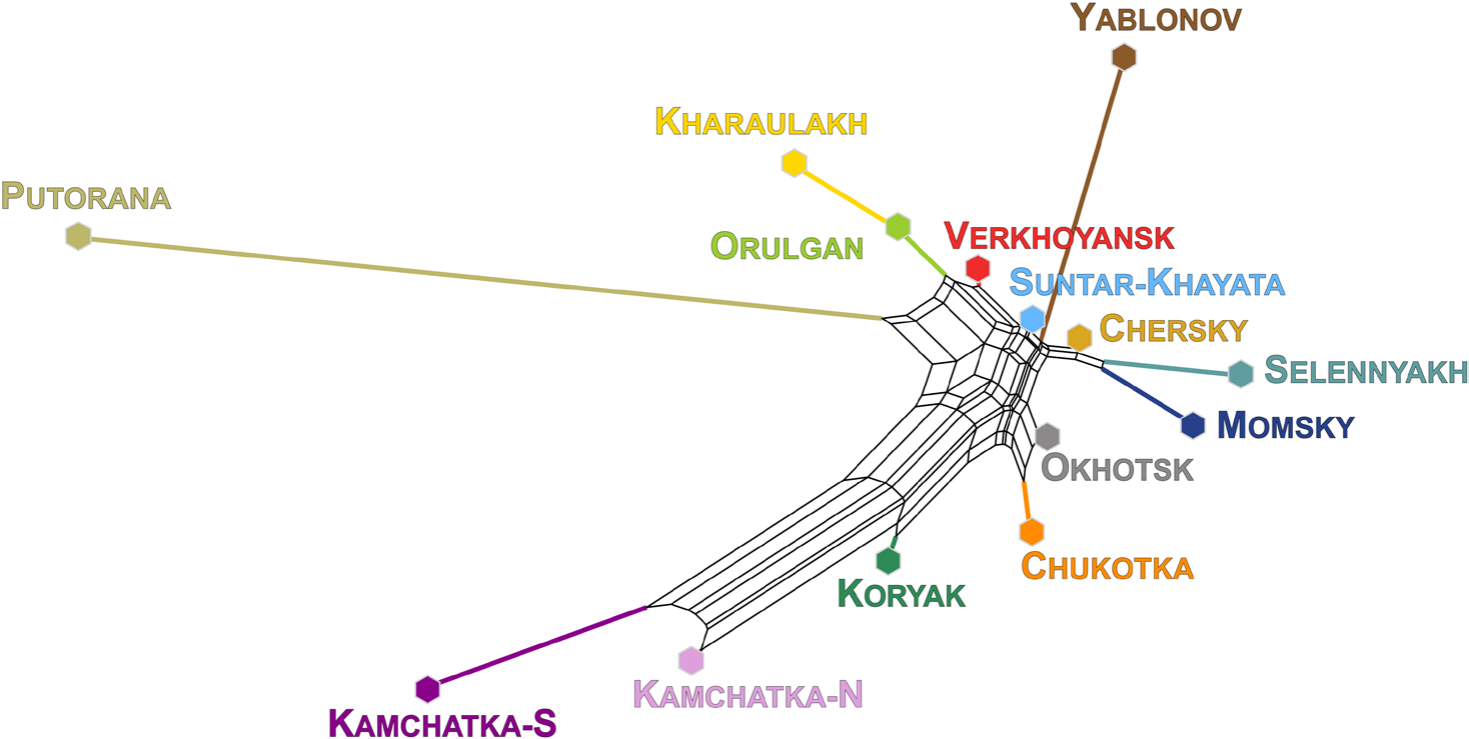
Neighbor‐net network of *Ovis nivicola* populations based on pairwise *F*_ST_ genetic distances.

Based on pairwise *F*_ST_ distances, a Neighbor‐Net network was constructed (Figure 4). The populations were arranged largely in accordance with their geographic distribution. Kamchatka‐S formed a distinct group, with Kamchatka‐N and Koryak positioned on the connecting branches linking it to the rest of the network. Okhotsk and Chukotka clustered closely together, while the Yakutian snow sheep populations—Chersky, Suntar‐Khayata and Verkhoyansk—were located in close proximity to one another. From Chersky, a branch split leading to Momsky and Selennyakh, whereas Yablonov diverged from Suntar‐Khayata. Further branches separated Kharaulakh and Putorana, with Kharaulakh connected to the main network via Orulgan, supporting the admixed origin of the latter.

**FIGURE 5:**
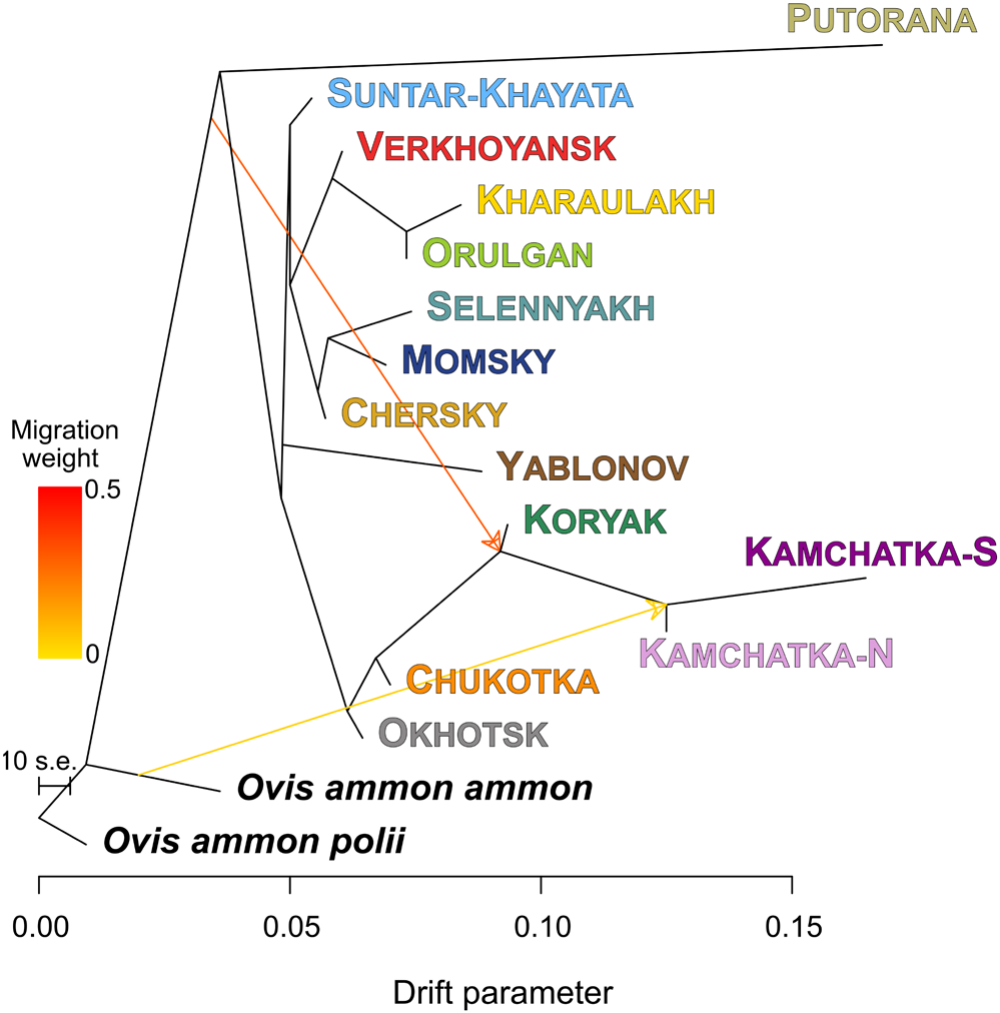
Maximum‐likelihood phylogenetic tree of *Ovis nivicola* populations reconstructed using TreeMix. It incorporates two migration events, with two *O. ammon* subspecies as the outgroup (topology observed in 94% of runs).

To infer population relationships and potential historical gene flow, we reconstructed a maximum‐likelihood phylogenetic tree using TreeMix (Figure 5). As an outgroup, we included the argali samples representing two subspecies, Altai ( *O. ammon ammon* ) and Pamir ( *O. ammon polii* ). To determine the optimal number of migration events, TreeMix was run with migration parameters ranging from 0 to 10. Based on the results using the R package OptM, the optimal model included two migration events (Figure S4). The first migration event originated from a common ancestor of all *O. nivicola* populations and was directed toward Koryak (observed in 96% of runs) (Figure S5). When two migration events were allowed, the second migration originated from *O. ammon ammon* and targeted the Kamchatka‐S/Kamchatka‐N branch (observed in 94% of runs) (Figure 5). The first migration event suggests that Koryak has a complex origin; although it is relatively close to Kamchatka, it does not derive directly from it. This pattern was also evident in the mitochondrial DNA analysis (Dotsev et al. 2021), where haplotypes from the Koryak Mountains clustered more closely with Yakutian samples and were distant from those of Kamchatka. The second migration event indicates that the Kamchatka population was the first to diverge from the common ancestor of all studied populations, thus representing a lineage with the longest independent evolutionary history. This finding is likewise consistent with mitochondrial DNA data.

When the number of migration events was increased, the tree topology became unstable. The most frequently observed third migration, occurring in 40% of runs, originated from the common ancestor and was directed toward the Kharaulakh/Orulgan branch (Figure S6).

The calculated residual fits (Figures S7–S9) showed low standard errors across populations, particularly for the model with two migration events (*m* = 2).

### 3.3 Within‐Population Genetic Diversity

Genetic diversity varied essentially among the populations (Table 2). The highest *H*_*O*_ value was detected in the Suntar‐Khayata population (0.220 ± 0.006), followed by Verkhoyansk (0.212 ± 0.006) and Chersky (0.209 ± 0.006), whereas the Putorana population showed extremely low heterozygosity (0.010 ± 0.002), consistent with its small population size and isolation. The *A*_*R*_ index followed a similar pattern, ranging from 1.616 ± 0.012 in Suntar‐Khayata to only 1.037 ± 0.006 in Putorana. In most populations, *F*_IS_ coefficients were close to zero or slightly positive, indicating near‐random mating, although elevated values were observed in Kamchatka‐S (0.086) and Putorana (0.145). Overall, the Yakutian populations (Suntar‐Khayata, Verkhoyansk and Chersky) exhibited the highest levels of genetic diversity, while the isolated Putorana group displayed markedly reduced variation.

**FIGURE 6:**
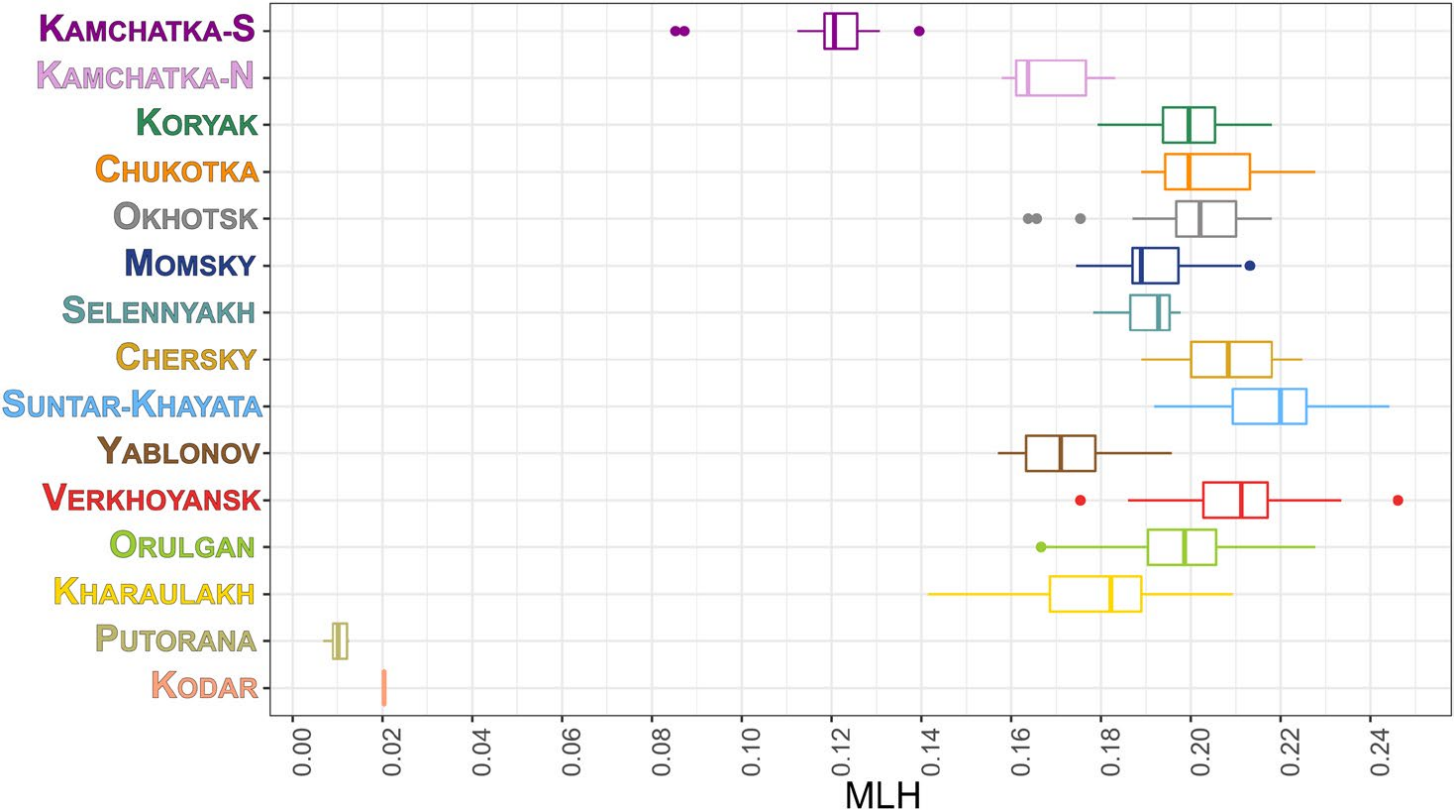
Distribution of individual multilocus heterozygosity (MLH) across *Ovis nivicola* populations shown as boxplots.

To assess genetic diversity further, we analyzed MLH values across populations (Figure 6). Unlike the main diversity metrics (Table 2) that did not allow a meaningful assessment of the single Kodar sample, MLH made it possible to visualize its heterozygosity relative to other groups. The Kodar individual exhibited extremely low heterozygosity, only slightly higher than that observed in the highly isolated Putorana population. Overall, the boxplots highlight pronounced differences among populations, with the Yakutian groups showing the highest heterozygosity and the isolated Putorana and Kodar populations the lowest.

## 4 Discussion

Herein we present the first comprehensive molecular–genetic investigation of the snow sheep ( *O. nivicola* ), including representatives of all major described subspecies. By integrating broad‐scale genomic analyses with a review of taxonomic history and changing perspectives on subspecies validity, we provide a unified framework for understanding evolutionary and taxonomic relationships within this species. In the following sections, we summarize the historical taxonomy of snow sheep subspecies, review their recognition in the scientific community, and present our genome‐wide SNP–based evaluation of each.

### 4.1 Kamchatkan Snow Sheep ( *Ovis nivicola nivicola* , Eschscholtz 1829)

The first records of wild sheep on Kamchatka date back to the second half of the 18th century and were made by Georg Wilhelm Steller (Nasonov 1923). In 1829, a specimen from Kamchatka was used by Johann Friedrich Eschscholtz to describe a new species, the snow sheep. With the subsequent development of intraspecific taxonomy, this population was designated as the nominal subspecies, *O. n. nivicola*. The taxonomic validity of this subspecies has been consistently recognized in major systematic treatments (Nasonov 1923; Tsalkin 1951; Sokolov 1959; Heptner et al. 1961; Valdez 1982; Zheleznov‐Chukotsky 1994; Chernyavsky 2004; Fil and Mosolov 2010; Damm and Franco 2014). Our findings demonstrate significant differentiation between Kamchatkan snow sheep and other populations (Figures 3A,C, 4, 5), thereby providing molecular genetic support for previous taxonomic assessments. Additionally, we found evidence of genetic admixture between individuals from the northern part of the subspecies' range and the population inhabiting the Koryak Mountains (Figure 3B and Table 2). Genetic diversity indices in the Kamchatka snow sheep were lower than in most other groups, being higher only than those of the isolated Putorana and Kodar populations (Table 3 and Figure 6).

**TABLE 3:** Genetic diversity parameters in the studied snow sheep populations.

Populations	*n*	*H*_*O*_	_*U*_ *H*_*E*_	*F*_IS_ [95% CI]	*A*_*R*_
Kamchatka‐S	18	0.120 ± 0.006	0.134 ± 0.006	0.086 [0.063; 0.109]	1.358 ± 0.014
Kamchatka‐N	6	0.170 ± 0.007	0.179 ± 0.006	0.044 [0.015; 0.073]	1.482 ± 0.016
Koryak	37	0.201 ± 0.006	0.204 ± 0.006	0.013 [0.001; 0.025]	1.549 ± 0.013
Chukotka	7	0.206 ± 0.007	0.207 ± 0.006	0.001 [−0.015; 0.035]	1.562 ± 0.015
Okhotsk	22	0.201 ± 0.006	0.210 ± 0.006	0.034 [0.019; 0.049]	1.583 ± 0.013
Momsky	12	0.194 ± 0.006	0.196 ± 0.006	0.004 [−0.014; 0.022]	1.543 ± 0.014
Selennyakh	7	0.191 ± 0.007	0.187 ± 0.006	−0.014 [−0.039; 0.011]	1.507 ± 0.015
Chersky	19	0.209 ± 0.006	0.216 ± 0.006	0.029 [0.014; 0.044]	1.601 ± 0.013
Suntar‐Khayata	45	0.220 ± 0.006	0.222 ± 0.006	0.009 [−0.001; 0.019]	1.616 ± 0.012
Yablonov	6	0.175 ± 0.007	0.175 ± 0.007	0.006 [−0.025; 0.037]	1.439 ± 0.015
Verkhoyansk	38	0.212 ± 0.006	0.214 ± 0.006	0.011 [0; 0.022]	1.592 ± 0.012
Orulgan	72	0.200 ± 0.006	0.202 ± 0.006	0.013 [0.005; 0.021]	1.557 ± 0.013
Kharaulakh	41	0.180 ± 0.006	0.183 ± 0.006	0.018 [0.005; 0.031]	1.486 ± 0.014
Putorana	6	0.010 ± 0.002	0.013 ± 0.002	0.145 [0.010; 0.280]	1.037 ± 0.006

*Note: A*_*R*_, allelic richness; *F*_IS_ [95% CI], inbreeding coefficient with 95% confidence interval; *H*_*O*_, observed heterozygosity; *n*, sample size; _*U*_ *H*_*E*_, unbiased expected heterozygosity.

### 4.2 Putorana Snow Sheep ( *Ovis nivicola borealis* , Severtzov 1873), Synonym Norilsk Snow Sheep

In 1873, Nikolai Severtzov described a specimen from the Putorana Plateau as morphologically intermediate between *O. nivicola* and *O. ammon* , though more closely resembling the former (Nasonov 1923). Although some studies failed to detect significant morphological differentiation, the Putorana population retained its classification as a distinct subspecies (*O. n. borealis*), primarily due to its geographic isolation (> 1000 km from the nearest populations) (Tsalkin 1951; Heptner et al. 1961). Our molecular genetic analyses revealed significant differentiation between this population and all other recognized snow sheep groups (Figure 4). This distinction was supported by a relatively high minimum genetic divergence (*F*_ST_ ≥ 0.381) with respect to other populations, as well as the presence of a private ancestral component at *K* = 4 in the ADMIXTURE analysis (Figure 3C). Notably, the Putorana subspecies exhibits the lowest genetic diversity across the species' range, indicating potential conservation concerns (Table 3 and Figure 6).

### 4.3 Okhotsk Snow Sheep ( *Ovis nivicola* *alleni*, Matschie 1907), Synonyms Kolyma Snow Sheep and Taygonos Snow Sheep

In 1903, Joel Asaph Allen reported discovering a wild sheep skull on the Taygonos Peninsula (the northern coast of the Sea of Okhotsk). Initially attributing this find to *O. n. nivicola*, he later reconsidered in favor of *O. n. borealis*. The following year (1904), J. Allen proposed classifying this specimen as a distinct species. Ultimately, in 1907, Paul Matschie formally described this population as a distinct subspecies—*O. n. alleni*. However, this subspecific classification remained contentious in subsequent systematic revisions. Nasonov (1923) recognized the validity of this subspecies and further suggested that populations from the Stanovoy Range might also belong to it. However, he noted that available material was insufficient for definitive confirmation. Tsalkin (1951), Heptner et al. (1961) and Zheleznov‐Chukotsky (1994) reached largely similar conclusions and included the mountains of the Taygonos Peninsula, Stanovoy Range, Dzhugdzhur and the southwestern Kolyma Mountains within the subspecies' range. Different taxonomic interpretations were proposed by Revin et al. (1988), Chernyavsky (2004) and Damm and Franco (2014), who did not recognize *O. n. alleni* as a valid subspecies, instead assigning populations throughout this range to the Yakutian subspecies (*O. n. lydekkeri*). Our research demonstrated that the Okhotsk snow sheep, together with the Chukotka population, formed a distinct PCA cluster and maintained a private ancestral component in the ADMIXTURE analysis (*K* = 8) (Figure 3C). Notably, their *F*_ST_‐based genetic distances relative to the Yakutian populations were rather low: 0.057 to the Chersky population and 0.058 to Suntar‐Khayata, respectively (Table S1 and Figure 4). The comparative analysis of samples from the Kolyma Mountains and Stanovoy Range (Yablonov) revealed significant genetic differentiation (Figure 5), precluding their classification as a single group. Considering all the above evidence, our results support the recognition of the Okhotsk snow sheep as a valid subspecies—*O. n. alleni*. The range of this subspecies comprises the Kolyma Mountains, Anadyr Plateau, and Chukotka Mountains.

### 4.4 Yakutian Snow Sheep ( *Ovis nivicola* *lydekkeri*, Kowarzik 1913)

Early records of wild sheep in Yakutia region were documented by Alexander von Bunge (1887) and Ivan D. Chersky (1891), who classified them as *O. borealis* and *O. nivicola* , respectively (Nasonov 1923). The first specimen from the Verkhoyansk Range was described by Richard Lydekker (1901), who did not distinguish it morphologically from the Putorana population. Several years later, Rudolf Kowarzik (1913) formally described this population as a subspecies, designating it *O. n. lydekkeri* in honor of Richard Lydekker (Nasonov 1923). Subsequently, the validity of this subspecies has never been challenged by researchers and disagreements have concerned only its range. Some taxonomists have attributed to it only the populations inhabiting mountains of Yakutia, that is, Verkhoyansk, Suntar‐Khayata, Momsky and Chersky ranges (Tsalkin 1951; Zheleznov‐Chukotsky 1994), while others have defined a larger distribution, including neighboring regions like Chukotka and Khabarovsk Krai (Revin et al. 1988; Chernyavsky 2004; Damm and Franco 2014).

In the current study, given the vast distribution range, we divided the snow sheep from the Yakutian mountains into seven separate populations according to their associated mountain ranges, that is, Central Verkhoyansk, Suntar‐Khayata, Chersky, Momsky, Selennyakh, Orulgan, and Kharaulakh (Figures 1A and 2A and Figure S1A,B). In these groups, four ancestral components, which corresponded to the Momsky, Central Verkhoyansk, Kharaulakh, and Suntar‐Khayata populations, were revealed by the ADMIXTURE analysis (Figure 3C). Notably, the Suntar‐Khayata component was shared with the Yablonovy snow sheep population from the Stanovoy Range. Furthermore, the Orulgan Range population exhibited an admixture of the Kharaulakh and Central Verkhoyansk components, a finding that was also supported by the results of f_3_‐statistics (Table 2). Therefore, our findings indicate that the Yakutian snow sheep consists of several genetically distinct populations. Further research is necessary to determine whether they warrant division into separate subspecies or should be maintained as a single taxonomic group.

### 4.5 Yablonov Snow Sheep ( *Ovis nivicola* *potanini*, Nasonov 1915), Synonym Stanovoy Range Snow Sheep

Yablonov snow sheep (*O. n. potanini*) was described in 1915 by Nasonov and Dorogostaisky (1915) “from material obtained during a 1914 faunal survey of the Yablonovy Ridge, an expedition organized by the Russian Imperial Academy of Sciences and led by V. C. Dorogostaisky.” It is important to note that in the early 20th century, the orographic terminology of Siberia was insufficiently developed. Nasonov and Dorogostaisky considered the term “Yablonovy” to be synonymous with the Stanovoy Range. Today, there is a clear understanding that these are distinct mountain ranges; snow sheep inhabit the Stanovoy Range and are absent from the Yablonovy (Heptner et al. 1961). Based on the coordinate data from the expedition description, the material was likely collected on the territory of the Stanovoy Range. However, in the current study, we retained the historical name “Yablonov sheep” to ensure clarity when discussing this specific historical context and literature. Nasonov and Dorogostaisky (1915) noted that this group of animals is most closely related to *O. n. lydekkeri*, from which it differs in lacking white spots on the flanks above the groin. It is distinguished from *O. n. alleni* by its horn curvature and from *O. n. nivicola* by the presence of white spots on its flanks (Nasonov and Dorogostaisky 1915). In 1951, Tsalkin considered the designation of a distinct subspecies to be unjustified, concluding that the differences in horn curvature from *O. n. alleni* were too weak to warrant any systematic importance. This conclusion was supported by subsequent taxonomic works (Heptner et al. 1961; Chernyavsky 1984; Revin et al. 1988; Damm and Franco 2014), and the sheep from the Yablonovy and Stanovoy Ranges were consequently classified within the Okhotsk subspecies (*O. n. alleni*). However, some researchers considered *O. n. potanini* to be a distinct subspecies (Bunch et al. 2006), noting the presence of a white patch behind the shoulders compared to *O. n. alleni* (Sokolov 1959).

This study demonstrates that the so‐called Yablonov snow sheep is genetically distinct from *O. n. alleni* (*F*_ST_ = 0.169) and is most closely related to the population from the Suntar‐Khayata Range (*F*_ST_ = 0.126) (Table S1 and Figure 4), with which it shares a distinct ancestral component in the ADMIXTURE analysis (Figure 3C). These results support the hypothesis that this population represents a genetically distinct lineage and are consistent with the recognition of *O. n. potanini* as a valid subspecies. However, given the limited sample size available from the Stanovoy Range, additional sampling and genomic data are desirable to further evaluate its taxonomic status. Based on the clarified type locality, the historical taxon is more appropriately referred to as the Stanovoy snow sheep rather than the Yablonov snow sheep.

### 4.6 Koryak Snow Sheep ( *Ovis nivicola koriakorum* , Chernyavsky 1962)

In 1962, Chernyavsky (1962) studied a series of skulls and hides from the Koryak Mountains, leading him to conclude that this isolated population represents a well‐differentiated subspecies. The author noted that this form is characterized primarily by its relatively small size, as well as by several specific cranial proportions (Chernyavsky 1962). In terms of the length and massiveness of the horn cores and the basal horn thickness in males, the Koryak sheep occupies an intermediate position between *O. n. nivicola* and *O. n. lydekkeri*. In subsequent taxonomic studies, this subspecies has been generally recognized as valid (Zheleznov‐Chukotsky 1994; Fil and Mosolov 2010; Damm and Franco 2014), although some authors have synonymized it with the Yakutian subspecies (Revin et al. 1988). Here, the Koryak sheep formed a distinct PCA cluster (Figure 3A) and possessed a unique ancestral component in the ADMIXTURE analysis (Figure 3C). These results provide strong genetic support for its recognition as a separate subspecies.

### 4.7 Chukotkan Snow Sheep ( *Ovis nivicola* *tschuktschorum*, Zheleznov 1990)

The first references to the presence of wild sheep in Chukotka date back to the second half of the 19th century. Karl K. Neumann, in his 1871 report, noted that “wild sheep are distributed throughout the Chukotka land and reach the Arctic Ocean.” The occurrence of sheep in the mountains of the Chukotka Peninsula was also mentioned by Innokenty P. Tolmachoff in 1911 (Nasonov 1923). However, the limited availability of material at the time prevented researchers from definitively determining the taxonomic status of the Chukotkan sheep. Tsalkin (1951) suggested their affinity to the Yakutian subspecies, whereas Heptner et al. (1961) considered the possibility of Okhotsk sheep dispersing into Chukotka along the ridges of the Kolyma Mountains. In 1984, Chernyavsky (1984) proposed the distinctiveness of the Chukotkan sheep. In 1990, Zheleznov (1990) described it as a separate subspecies, *O. n. tschuktschorum*, highlighting notable morphological differences in this population. Although this subspecies has been recognized in several subsequent studies (Shackleton 1997; Krivoshapkin and Yakovlev 1999; Fil and Mosolov 2010), Chernyavsky (2004) argued that its designation was inconsistent with the International Rules of Zoological Nomenclature (CIPNZ 1905).

In a recent study based on genome‐wide SNP and mitochondrial DNA data (Dotsev et al. 2026), Chukotkan sheep were shown to cluster closely with *O. n. alleni* (Okhotsk sheep). Our current analysis, using the same Chukotka samples, is consistent with these findings by incorporating comparisons with a broader set of populations, including additional geographic and genetic diversity. Specifically, our PCA revealed overlapping clusters between these groups (Figure 3A), and ADMIXTURE analysis indicated a shared ancestral component, differing only by a minor contribution from Koryak ancestry in the Chukotkan sheep and from Yakutian (Momsky) ancestry in the Okhotsk sheep (Figure 3C). Additionally, the close relationship was supported by the low *F*_ST_ value between these two groups (Table S1). Based on these findings, we suggest that the Chukotkan sheep are more appropriately aligned with the Okhotsk sheep and classified as *O. n. alleni*.

### 4.8 Kodar Snow Sheep ( *Ovis nivicola kodarensis* , Medvedev 1994)

The Kodar sheep represents a small, isolated group of approximately 300 individuals inhabiting the Kodar Mountains. Prior to its description as a distinct subspecies by Medvedev (1994), this population was only rarely mentioned in taxonomic works (Revin et al. 1988). A comparative analysis using SNP markers was conducted in 2017, in which the Kodar population was contrasted with the Yakutian populations, suggesting its genetic distinctiveness (Medvedev et al. 2017). However, that study included only a single Kodar sample, and the number of available specimens has not increased since then due to the listing of this group in the regional Red Data Book, which restricts the collection of new material. We argue that such a limited dataset is insufficient to either confirm or refute the taxonomic status of the Kodar sheep. At the same time, it is important to note the extremely low genetic diversity observed in the single available sample (Figure 6), which most likely reflects the small census size and long‐term isolation of this population.

### 4.9 Kharaulakh Snow Sheep ( *Ovis nivicola* *ernsti*, Proposed)

The proposal to consider the snow sheep from the Kharaulakh Range as distinct from *O. n. lydekkeri* emerged following a genome‐wide SNP analysis of the Yakutian subspecies populations published in 2018 (Dotsev et al. 2018). This study demonstrated that the Kharaulakh sheep has an origin distinct from other groups within the subspecies, while the population from the Orulgan Range showed a mixed ancestry, sharing ancestral components with both the Kharaulakh and Central Verkhoyansk populations. Subsequently, in 2021, this taxonomic status was confirmed in a study utilizing a different type of genetic marker—mitochondrial DNA (Dotsev et al. 2021). This later work also revealed the uniqueness of the Kharaulakh population's mitochondrial lineage. The Orulgan population possessed haplotypes characteristic of both the Kharaulakh and Central Verkhoyansk groups, which were highly divergent from one another. This pattern further supports the hybrid origin of the Orulgan Range population. It is important to note that Kowarzik, who described *O. n. lydekkeri* in 1913, stated in a subsequent work that this subspecies inhabits the Yana River basin, while another form—*O. lenaensis*—inhabits the Lena River basin. However, he failed to provide a description for the latter. As early as 1923, Nasonov (1923) criticized this division, finding no substantial morphological differences between the two. He also argued that the separation along the Verkhoyansk and Kharaulakh Ranges, as proposed by Kowarzik, could not serve as a valid subspecies boundary. Nasonov (1923) reasoned that these mountain ranges are not high, particularly the Kharaulakh Range, and that movement between their slopes would have been possible. Consequently, the Kharaulakh sheep was subsequently considered part of *O. n. lydekkeri* in all further taxonomic treatments. Our present study is consistent with the results of previous genetic studies (Dotsev et al. 2018, 2021), now utilizing a significantly larger sample size. In our view, the Kharaulakh population represents descendants of a group of snow sheep that survived in a refugium near Tiksi Bay during the glaciation, when most of the Verkhoyansk Range was covered with ice and became uninhabitable. Following the retreat of the glaciers, the Kharaulakh population began to expand southward. At the same time, the Verkhoyansk Range was being colonized by populations from the south and in the Orulgan Range, a secondary contact occurred between the northern and southern lineages. This scenario explains the admixed genetic origin of the Orulgan population.

Based on our current findings, we formally propose the Kharaulakh snow sheep as a distinct subspecies, for which we suggest the scientific name *Ovis nivicola ernsti*. This name honors Academician Lev Konstantinovich Ernst (1929–2012) who was instrumental in initiating this research, served as the scientific advisor for several authors of this article, and after whom the institute where the primary genetic studies of snow sheep were conducted is named.

### 4.10 Patterns of Genetic Diversity Across the Species Range

Genetic diversity in snow sheep exhibited pronounced spatial patterns strongly associated with geographic position, habitat connectivity, and the degree of isolation. The highest levels of heterozygosity and allelic richness were detected in the central Yakutian populations, particularly Suntar‐Khayata, Verkhoyansk, and Chersky. These mountain ranges form a network in the core of the species' distribution and likely maintain connectivity among neighboring populations over evolutionary time, thereby facilitating gene flow.

In contrast, the lowest levels of genetic diversity were observed in the Putorana population, which is separated from the nearest snow sheep populations by more than 1000 km and has an estimated population size of approximately 3500 individuals. This long‐term geographic isolation, together with its relatively small population size, has likely contributed to the exceptionally low levels of heterozygosity and allelic richness observed in this population. A similar pattern was observed for the Kodar population, although conclusions for this group remain preliminary because only a single individual was available for analysis.

Intermediate levels of diversity were found in the Kamchatka and Kharaulakh populations. The southern Kamchatka population, located on a peninsula with limited connectivity to mainland groups, showed reduced heterozygosity and a slightly elevated inbreeding coefficient, consistent with restricted gene flow. The Kharaulakh population also exhibited lower diversity than the remaining Yakutian groups. This pattern may reflect its peripheral position at the northern margin of the species' distribution, where reduced connectivity and smaller effective population sizes are expected. In addition, the distinct evolutionary history of this population, which may have persisted in a northern refugium during the Pleistocene, likely contributed to the comparatively lower genetic diversity observed in this population. Notably, the Orulgan Range population, situated at the contact zone between the Kharaulakh and Central Verkhoyansk lineages, exhibited both intermediate genetic diversity and significant admixture, consistent with the results of the f_3_‐statistics and ADMIXTURE analyses.

Overall, these results indicate that contemporary patterns of genetic diversity in snow sheep are shaped primarily by long‐term geographic isolation and historical connectivity among mountain systems rather than by geographic distance alone. Similar patterns have been reported in other mountain ungulates. Genome‐wide SNP analyses of thinhorn sheep ( *Ovis dalli* ) demonstrated that populations originating from different Pleistocene refugia differed markedly in genetic diversity, with reduced diversity in lineages that persisted in smaller, isolated refugia (Sim et al. 2016). Likewise, landscape genomic analyses of northern chamois ( *Rupicapra rupicapra* ) showed that historical climatic stability and landscape connectivity are major determinants of population structure and genetic diversity (Roques et al. 2026). Taken together, these comparisons suggest that the observed diversity gradient in snow sheep reflects the combined effects of Pleistocene history and long‐term geographic isolation among mountain systems.

### 4.11 Study Limitations and Future Prospects

A limitation of the present study is the use of a domestic sheep ( *O. aries* ) SNP array for genotyping wild snow sheep ( *O. nivicola* ). Such SNP‐chip data are not ideally suited for analyses that rely on dense genome‐wide marker coverage and local linkage disequilibrium, including estimation of effective population size from LD, detection of runs of homozygosity (ROH), and other haplotype‐based demographic analyses. These approaches generally require much higher marker density than was available after quality filtering.

Nevertheless, SNP arrays have been widely and successfully applied to investigate population structure, genetic differentiation, and admixture in wild ungulates. In the present study, all samples belonged to a single species and were genotyped using the same SNP panel, allowing consistent comparisons among populations. Furthermore, the major patterns of population structure were concordant across multiple independent analytical approaches (PCA, ADMIXTURE, pairwise *F*_ST_, Neighbor‐Net, TreeMix, and f_3_‐statistics), indicating that the retained SNP dataset provides sufficient resolution for the population genetic questions addressed here.

Future studies based on whole‐genome sequencing or species‐specific SNP panels will enable more detailed reconstruction of the demographic history of snow sheep, including changes in effective population size, patterns of historical gene flow, and the timing of divergence among major lineages. Increased sampling from poorly represented populations, particularly the Kodar population, will also be essential for resolving their evolutionary history and taxonomic status.

## 5 Conclusions

In this study, we performed a genome‐wide SNP analysis of all major snow sheep ( *O. nivicola* ) groups and investigated patterns of intraspecific genetic structure. Our results support the current subspecific designations for the Kamchatkan (*O. n. nivicola*), Putorana (*O. n. borealis*), Yablonov/Stanovoy (*O. n. lydekkeri*), Okhotsk (*O. n. alleni*), Yakutian (*O. n. lydekkeri*), Koryak (*O. n. koriakorum*), and Kharaulakh (*O. n. ernsti*, proposed) populations. The taxonomic status of the Kodar snow sheep requires further investigation with additional samples. The Chukotkan snow sheep is genetically consistent with *O. n. alleni*, sharing a common ancestral genetic component and showing only minor admixture from neighboring groups. Extremely low genetic diversity was detected in the Putorana and Kodar populations, although these estimates should be interpreted with caution due to limited sample sizes. Overall, the results provide new insights into the intraspecific genetic structure of snow sheep and offer a framework for future conservation and genomic studies.

## Author Contributions

**Arsen V. Dotsev:** conceptualization (lead), data curation (lead), formal analysis (lead), investigation (lead), software (lead), writing – original draft (lead). **Innokentiy M. Okhlopkov:** conceptualization (equal), resources (equal), supervision (equal). **Mikhail G. Bondar:** conceptualization (equal), resources (equal). **Dennis I. Litovka:** conceptualization (equal), resources (equal). **Tatiana E. Deniskova:** investigation (equal), methodology (equal), validation (equal). **Veronika R. Kharzinova:** investigation (equal), methodology (equal), validation (equal). **Michael N. Romanov:** supervision (equal), validation (equal), writing – review and editing (lead). **Taras P. Sipko:** investigation (equal), resources (equal). **Nikolai V. Mamaev:** investigation (equal), resources (equal). **Maria N. Semerikova:** investigation (equal), resources (equal). **Olga A. Koshkina:** investigation (equal), methodology (equal). **Neckruz F. Bakoev:** investigation (equal), methodology (equal). **Vugar A. Bagirov:** investigation (equal), supervision (equal). **Andrey A. Sitsko:** investigation (equal), resources (equal). **Dmitry G. Medvedev:** investigation (equal), resources (equal). **Alexander N. Kuksin:** investigation (equal), resources (equal). **Mohammad Hossein Moradi:** investigation (equal), methodology (equal). **Ali Esmailizadeh:** investigation (equal), methodology (equal). **Darren K. Griffin:** validation (equal), writing – review and editing (equal). **Henry Reyer:** investigation (equal), writing – review and editing (equal). **Klaus Wimmers:** investigation (equal), writing – review and editing (equal). **Gábor Mészáros:** investigation (equal), writing – review and editing (equal). **Johann Sölkner:** investigation (equal), writing – review and editing (equal). **Ivica Medugorac:** investigation (equal), writing – review and editing (equal). **Gottfried Brem:** supervision (equal), validation (equal). **Natalia A. Zinovieva:** conceptualization (equal), supervision (lead), writing – review and editing (equal).

## Funding

This study was supported by the Russian Science Foundation (Project No. 24‐44‐20011) and the Iran National Science Foundation (Project No. 4023205).

## Conflicts of Interest

The authors declare no conflicts of interest. The funders had no role in the design of the study; in the collection, analyses, or interpretation of the data; in the writing of the manuscript; or in the decision to publish the results.

## Supporting information


**Figure S1:** Principal component analysis (PCA) of genetic variation among *Ovis nivicola* populations (PC1–PC3). (A) Analysis including all populations. (B) Analysis including populations from Yakutia and the Stanovoy Range (Yablonov).
**Figure S2:** Cross‐validation error values from ADMIXTURE analyses used to infer the optimal number of genetic clusters (K).
**Figure S3:** Estimated individual ancestry proportions of *Ovis nivicola* populations inferred using ADMIXTURE for values of K ranging from 2 to 8.
**Figure S4:** Model selection for the number of migration events in TreeMix using OptM: (A) likelihood values across models; (B) Δm statistic indicating the optimal number of migration events.
**Figure S5:** Maximum‐likelihood phylogenetic tree of *Ovis nivicola* populations reconstructed using TreeMix, incorporating one migration event, with *Ovis ammon* set as the outgroup (topology observed in 96% of runs).
**Figure S6:** Maximum‐likelihood phylogenetic tree of *Ovis nivicola* populations reconstructed using TreeMix, incorporating three migration events, with *Ovis ammon* set as the outgroup (topology observed in 40% of runs).
**Figure S7:** Residual fit from the TreeMix analysis with one migration event (*m* = 1). Positive residuals indicate pairs of populations that are more closely related in the data than expected under the inferred tree.
**Figure S8:** Residual fit from the TreeMix analysis with two migration events (*m* = 2). Positive residuals indicate pairs of populations that are more closely related in the data than expected under the inferred tree.
**Figure S9:** Residual fit from the TreeMix analysis with three migration events (*m* = 3). Positive residuals indicate pairs of populations that are more closely related in the data than expected under the inferred tree.
**Table S1:** Pairwise *F*_ST_ values among *Ovis nivicola* populations based on genome‐wide SNP data.

## Data Availability

The PLINK genotyping files (Datasets 1 and 2) analyzed in this study have been deposited on Figshare and are available to reviewers via the private link: https://figshare.com/s/e7e5be7245f784834ca8. The datasets will be made publicly available with a permanent DOI immediately upon acceptance and publication of the article.
