# Supplementary material for: Unraveling the Evolutionary History of Snow Sheep (Ovis nivicola): Genome‐Wide Single Nucleotide Polymorphism Analysis Reveals Genetic Diversity and Clarifies Taxonomy
Source: Ecol Evol. 2026 Aug 14;16(8):e74182. doi: 10.1002/ece3.74182 (PMC13476151; doi:10.1002/ece3.74182)
Supplement: Supplementary file 1 [file ECE3-16-e74182-s001.zip › Appendix.docx]

**Appendix**

**Supplementary**

**Supplementary Figure S1.** Principal component analysis (PCA) of genetic variation among *Ovis nivicola* populations (PC1–PC3). **(A)** Analysis including all populations. **(B)** Analysis including populations from Yakutia and the Stanovoy Range (Yablonov).

**Supplementary Figure S2.** Cross-validation error values from ADMIXTURE analyses used to infer the optimal number of genetic clusters (K).

**Supplementary Figure S3.** Estimated individual ancestry proportions of *Ovis nivicola* populations inferred using ADMIXTURE for values of K ranging from 2 to 8.

**Supplementary Figure S4.** Model selection for the number of migration events in TreeMix using OptM: (A) likelihood values across models; (B) Δm statistic indicating the optimal number of migration events.

**Supplementary Figure S5.** Maximum-likelihood phylogenetic tree of *Ovis nivicola* populations reconstructed using TreeMix, incorporating one migration event, with *Ovis ammon* set as the outgroup (topology observed in 96% of runs).

**Supplementary Figure S6.** Maximum-likelihood phylogenetic tree of *Ovis nivicola* populations reconstructed using TreeMix, incorporating three migration events, with *Ovis ammon* set as the outgroup (topology observed in 40% of runs).

**Supplementary Figure S7.** Residual fit from the TreeMix analysis with one migration event (*m* = 1). Positive residuals indicate pairs of populations that are more closely related in the data than expected under the inferred tree.

**Supplementary Figure S8.** Residual fit from the TreeMix analysis with two migration events (*m* = 2). Positive residuals indicate pairs of populations that are more closely related in the data than expected under the inferred tree.

**Supplementary Figure S9.** Residual fit from the TreeMix analysis with three migration events (*m* = 3). Positive residuals indicate pairs of populations that are more closely related in the data than expected under the inferred tree.

**Supplementary Table S1.** Pairwise *F*_ST_ values among *Ovis nivicola* populations based on genome-wide SNP data.

| **Populations** | **1** | **2** | **3** | **4** | **5** | **6** | **7** | **8** | **9** | **10** | **11** | **12** | **13** | **14** |
| --- | --- | --- | --- | --- | --- | --- | --- | --- | --- | --- | --- | --- | --- | --- |
| 1. KAMCHATKA_S | 0 |  |  |  |  |  |  |  |  |  |  |  |  |  |
| 2. KAMCHATKA_N | 0.136 | 0 |  |  |  |  |  |  |  |  |  |  |  |  |
| 3. KORYAK | 0.248 | 0.117 | 0 |  |  |  |  |  |  |  |  |  |  |  |
| 4. CHUKOTKA | 0.354 | 0.205 | 0.109 | 0 |  |  |  |  |  |  |  |  |  |  |
| 5. OKHOTSK | 0.316 | 0.196 | 0.113 | 0.036 | 0 |  |  |  |  |  |  |  |  |  |
| 6. MOMSKY | 0.392 | 0.268 | 0.189 | 0.146 | 0.104 | 0 |  |  |  |  |  |  |  |  |
| 7. SELENNYAKH | 0.417 | 0.284 | 0.202 | 0.171 | 0.122 | 0.103 | 0 |  |  |  |  |  |  |  |
| 8. CHERSKY | 0.339 | 0.222 | 0.141 | 0.091 | 0.057 | 0.054 | 0.066 | 0 |  |  |  |  |  |  |
| 9. SUNTAR_KHAYATA | 0.307 | 0.204 | 0.136 | 0.086 | 0.058 | 0.083 | 0.086 | 0.027 | 0 |  |  |  |  |  |
| 10. YABLONOV | 0.451 | 0.339 | 0.239 | 0.206 | 0.169 | 0.206 | 0.226 | 0.149 | 0.126 | 0 |  |  |  |  |
| 11. VERKHOYANSK | 0.337 | 0.231 | 0.167 | 0.123 | 0.095 | 0.111 | 0.111 | 0.055 | 0.044 | 0.166 | 0 |  |  |  |
| 12. ORULGAN | 0.359 | 0.269 | 0.199 | 0.166 | 0.136 | 0.154 | 0.15 | 0.097 | 0.092 | 0.196 | 0.048 | 0 |  |  |
| 13. KHARAULAKH | 0.41 | 0.324 | 0.248 | 0.218 | 0.187 | 0.212 | 0.202 | 0.152 | 0.141 | 0.254 | 0.101 | 0.039 | 0 |  |
| 14. PUTORANA | 0.645 | 0.642 | 0.443 | 0.543 | 0.426 | 0.505 | 0.583 | 0.427 | 0.381 | 0.599 | 0.401 | 0.415 | 0.467 | 0 |
